# eHealth Literacy: In the Quest of the Contributing Factors

**DOI:** 10.2196/ijmr.4749

**Published:** 2016-05-25

**Authors:** Sofia Xesfingi, Athanassios Vozikis

**Affiliations:** ^1^ University of Piraeus Department of Economics University of Piraeus Piraeus Greece

**Keywords:** eHealth literacy, health information, Internet, demographic factors, life-style habits

## Abstract

**Background:**

Understanding the factors that influence eHealth in a country is particularly important for health policy decision makers and the health care market, as it provides critical information to develop targeted and tailored interventions for relevant patient–consumer segments, and further suggests appropriate strategies for training the health illiterate part of the population.

**Objective:**

The objective of the study is to assess the eHealth literacy level of Greek citizens, using the eHealth Literacy Scale (eHEALS), and further explore the factors that shape it and are associated with it.

**Methods:**

This empirical study relies on a unique sample of 1064 citizens in Greece in the year 2013. The participants were requested to answer various questions about their ability to solve health-related issues using the Internet, and to provide information about their demographic characteristics and life-style habits. Ordered logit models were used to describe a certain citizen’s likelihood of being eHealth literate.

**Results:**

The demographic factors show that the probability of an individual being eHealth literate decreases by 23% (*P*=.001) when the individual ages and increases by 53% (*P*<.001) when he or she acquires higher level of education. Among the life-style variables, physical exercise appears to be strongly and positively associated with the level of eHealth literacy (*P*=.001). Additionally, other types of technology literacies, such as computer literacy and information literacy, further enhance the eHealth performance of citizens and have the greatest impact among all factors.

**Conclusions:**

The factors influencing eHealth literacy are complex and interdependent. However, the Internet is a disruptive factor in the relationship between health provider and health consumer. Further research is needed to examine how several factors associate with eHealth literacy, since, the latter is not only related to health care outcomes but also can be a tool for disseminating social inequalities.

## Introduction

Health literacy has been identified as a public health goal for the twenty-first century and a significant challenge in health education. Trending toward a more consumer-centric health care system as part of an overall effort to improve the quality of health care and to reduce health care costs, it is important that services and training be provided so that the health care consumer could take a more active role in health care-related decisions [1]. Despite the concerns regarding the quality of online health information [2], the advent of the Internet has dramatically changed the landscape of health information, as recent estimates document that more than 80% of the Internet users search for health-related information online [3,4]. According to a recent Pew Internet Research [5] study on health, Internet, and mobile phones, “80% of Internet users, or 59% of U.S. adults, look online for health information” and “17% of cell phone owners, or 15% of adults, have used their phone to look up health or medical information”. Another study [6] estimates that 75 million people will use their mobile phones in 2014 to access health information.

With the tremendous growth of available information, users face challenges in searching, locating, evaluating, and effective use of the health-related information available on the Internet, as data safety remains one of the most commonly identified barrier with respect to the effective use of information available on the Web [1,7]. Despite these perils, studies have showed that health consumers increasingly use the Internet not only for information but also for communicating with peers and health professionals, and purchasing health products and services [8,9].

Recently, a subfield within medical informatics has emerged that develops information and communication technology tools, and applications for use in health care, particularly that of eHealth, that is, the ability of the individuals in searching, analyzing, and processing information from the Internet in order to address or solve health-related issues [10].

Among the first studies in the field is the seminal study of Norman and Skinner [11], which examines, in a systematic way, attributes that contribute to eHealth literacy. The authors state that eHealth literacy could be defined by a set of factors such as a person’s ability to present the health issue, educational background, health status at the time of the eHealth encounter, motivation for seeking the information, and the technologies used, and aims to empower individuals and enable them to fully participate in health decisions informed by eHealth resources. Numerous subsequent studies have investigated the relationship between eHealth literacy and various, mainly demographic, factors.

Our research study contributes to the aforementioned vein of literature and brings evidence on the factors that influence the eHealth literacy in Greece, where, lately, government policies were focused on enabling the access to the Internet for a large part of population.

We focused on Greece as 8 out of 10 Internet users there searched the Internet seeking health information [12]. This is a surprisingly high rate, given the low penetration of Internet in Greece [13]. A recent study [12] identified and explained the reasons for the slower than anticipated growth of Internet use in Greece. A series of factors hindering e-services adoption were identified, such as: (1) limited commercial trust and user concerns for transactions security, (2) factors connected with social background, (3) low quality of available Greek electronic services, (4) intellectual property rights and privacy issues, and (5) complex or time consuming processes. Furthermore, according to the OECD health data [14], Greece has demographics that could constitute a serious issue for the future, such as low birth rate and population distribution. At the same time, Greeks are on severe economic crisis and an elevated prevalence of certain diseases is already reported [15].

Therefore, we first constructed an index for the measurement of eHealth literacy, enriching and adapting the Norman and Skinner [16] eHealth Literacy Scale and using unique survey data from a sample of 1064 individuals for the year 2013. The marking of the eHealth literacy index is based on the answers of the interviewees on eight questions about a user’s ability in searching, analyzing, and processing information from the Internet in order to address or solve health-related issues. Next, we estimated the effect of various demographic, life-style factors and levels of technology literacy on the users’ eHealth performance.

The novelty of our study lies in, first, investigating an important question for health policy implications for Greece—there is no prior study in this subject matter. Second, we include a variety of life style factors that no other existing relating study has included so far—the related literature offers piece-meal approach (eg, some studies examine only the relation between eHealth and smoking, while others focus on eHealth age effects). Third, with our econometric approach (logit model) we were able to assess the effect of the covariates on different classes (1: low; 2: fair; 3: medium; 4: high) of eHealth literacy of citizens.

Our results demonstrate the important impact of the age and education level as well as that of physical exercise on eHealth literacy. Other types of technology literacy, such as computer literacy and information literacy, further enhance the eHealth performance of citizens and have the greatest impact among all factors.

## Methods

This section discusses the survey data, the modified eHealth literacy index, and presents the selection of the estimation method.

### Data

This empirical analysis relies on Web- and interview-based data obtained from a sample of 1064 citizens in Greece for the year 2013, using the Convenience Sampling Technique, that is, a nonprobability sampling technique where the subjects are selected due to their convenient accessibility and proximity to the researcher, that is, they are easiest to recruit for the study. The participants were requested to answer various questions about their ability to solve health-related issues using information from the Internet. The dependent variable, the eHealth literacy index, is defined as the ability of a certain individual to seek, find, understand, and appraise health information from electronic resources and apply that knowledge to address or solve a health problem, according to Norman and Skinner [16]. Table 1 presents the components’ marking-evaluation, based on which the eHealth literacy index is constructed. Each component was measured on a 5-grade scale so that the total summary of the eHealth literacy index ranges from 8 to 40 grades.

**Table 1:** Description and share of the components of the eHealth literacy index.

Variable	Percentage
I know what health resources are available on the Internet	11.6%
I know where to find helpful health resources on the Internet	12.2%
I know how to find helpful health resources on the Internet	13.3%
I know how to use the Internet to answer my health questions	13.4%
I know how to use the health information I find on the Internet to help me	13.3%
I have the skills I need to evaluate the health resources I find on the Internet	12.7%
I can differentiate high-quality health resources from low-quality health resources on the Internet	12.5%
I feel confident in using information from the Internet to make health decisions	11.0%

Further, the users were asked to provide information about their demographic characteristics and life-style habits (Table 3). Various demographic factors were included in the questionnaire, such as gender, age, marital status, education, and income, grouped according to Hellenic Statistical Authority classification. More specifically, demographic variables were grouped as follows: Gender: 0 for male and 1 for female; Age: 1 for ages 15–24 years, 2 for 25–39 years, 3 for 40–54 years, 4 for 55–64 years, 5 for 65–79 years, and 6 for >80 years; Marital Status: 1 for single, 2 for married, 3 for divorced, 4 for separated, and 5 for widow; Education: 1 for primary school, 2 for high school (first 3 years), 3 for technical education, 4 for high school (last 3 years), 5 for post-high school (excluding university), 6 for university, 7 for Masters, and 8 for PhD; Income: 1 for <€750, 2 for €751–1100, 3 for €1101–1450, 4 for €1451–1800, 5 for €1801–2200, 6 for €2201–2800, 7 for €2801–3500, 8 for >€3501.

Additionally, they were requested to answer whether they smoke or not, whether they workout more than once per week, and whether they consume alcohol on a regular basis.

Finally, the participants were invited to evaluate their skills related to computer and information literacy. The former, measures the skills of the participant regarding the use of computers, that is, use of search engines, sending e-mails, uploading messages on forums, use of the Internet for chatting, or construction of Web pages, while the latter measures the degree of frequency of relying on Internet search as a primary source of health-related issues and the importance of accessing the internet in order to find health-related sources.

### Model

The likelihood of a certain user (citizen–patient) being eHealth literate (able to search, analyze, and process information from the Internet in order to address or solve health-related issues), can be described by an ordered logit model defined as follows:

Pr(Y=c|Xi)=F(X_i_β),

where the endogenous variable Y is the degree of eHealth literacy and takes values from 1 to 4 (c) in accordance with the aforementioned abilities (1 for low, 2 for fair, 3 for enough, 4 for high); F is the standard logistic cumulative distribution function and Χ_i_ is a set of covariates defined as:

X_i_ β=β_0_+ β_1_Gender_i_+ β_2_Age_i_+ β_3_ Marital Status_i_+ β_4_Education_i_+β_5_Income_i_+ β_6_Smoking_i_+ β_7_Exercise_i_+ β_8_Alcohol_i_+ β_9_CI_i_+ β_10_IL_i_

where the first five variables consist the demographic factors (set D): Gender is a dummy variable that takes the values 0 and 1 if the participant is male and female respectively; Age is the age of the participants clustered as follows: class 1 (15–24), class 2 (25–39), class 3 (40–54), class 4 (55–64), class 5 (65–79), class 6 (>80 years old); Marital Status represents whether a participant is single (1), married (2), divorced (3), separated (4) or widow (5); Education is the level of education of each participant ranging from primary school (1) to PhD (8); Income is the income level of the participants clustered in eight groups (refer preceding discussion about classes’ classification).

The next three variables form the life-style set (set L) and are: Smoking is a dummy variable and represents whether the participants are smokers or not; Exercise is a dummy variable that takes the value 0 if the participant is not exercising more than once per week, otherwise is 1; Alcohol is a dummy variable and takes the value 0 if the participant is not drinking on a regular basis, otherwise is 1.

Finally, we also included technology related literacy covariates, namely CL, which captures the computer literacy of each participant and ranges from (0) for no knowledge to (2) for high knowledge, and IL is the information literacy of the participant and takes the values (1), (2), and (3) for low, fair, and high knowledge (refer preceding discussion about classes’ classification).

The selection of the variables in Χ_i_ set can be justified by relevant studies. More specifically, the demographic variables of age and education are documented in the studies of Baker et al. [17], Petch et al. [18], Watkins and Xie [19]; while Schwartz et al. [20], Andreasen et al. [21], Rudd et al. [22], and Veenhorf et al. [23], along with the variables of age and education, take into account the variable of income. Further, the variable of gender is explored in the study of Norman and Skinner [16]. The life-style factors, such as smoking, are mentioned in the study of Bodie and Dutta [24]. Finally, technology literacy is included in a handful of studies [11,24,25].

The model only applies to data that meet the proportional odds assumption. Suppose that the proportions of members of the statistical population who would answer Y=1, Y=2, Y=3, Y=4, and Y=5 are, respectively, p1, p2, p3, p4, and p5; then the logarithms of the odds (not the logarithms of the probabilities) of answering in certain ways are as shown in Table 2.

**Table 2:** The proportional odds assumption

Probabilities	Logarithms of odds
Y=1,	log [p1/(p2+p3+p4+p5)], 0
Y=1 or Y=2,	log [(p1+p2)/(p3+p4+p5)], 1
Y=1, Y=2 or Y=3,	log [(p1+p2+p3)/(p4+p5)], 2
Y=1, Y=2, Y=3 or Y=4,	log [(p1+p2+p3+p4)/p5], 3

The proportional odds assumption is that the number added to each of these logarithms to get the next is the same in every case. In other words, these logarithms form an arithmetic sequence.

## Results

Before presenting our estimates of the model, we first show some descriptive statistics in Table 3.

As shown in Table 3, our sample participants have fair level of eHealth literacy. Further, half of the participants are men, while the majority of the interviewers are between the age of 25 and 39 years, and belong to middle income class. Furthermore, participants appear to lead healthy life-style, as they do not smoke or consume alcohol daily and workout more than once a week.

The correlation between the dependent variable of eHealth literacy and all the other factors (independent variables) are presented in Table 4.

As Table 4 shows, the two types of technology literacy, computer and information literacy, are highly related with eHealth literacy (0.46 and 0.45, respectively). These two variables are also positively related with each other. Further, age, education, and exercise are also strongly related with eHealth literacy (-0.29, 0.41, and 0.20, respectively).

The odds ratios for all specifications are presented in Table 5. One can read the odds ratios as follows: if the odd ratio, a, is bigger than 1 (a>1), then the probability of a user being health literate, (ie, Y_it_=4; maximum level of eHealth literacy), increases by (a-1)*100%, whereas the probability decreases by (1-a)*100%, if the odds ratio is smaller than 1 (a<1).

Columns (1) and (2) present estimates of the model, where only the demographic (D) and literacy factors (C) are included. Next, columns (3) and (4) show estimates of the model, where only the indicators of the participants’ life-style (L) and literacy are included. Finally, columns (5) and (6) present estimates, where the full set of covariates (X) are included.

As Table 5 shows, among the demographic factors (D) presented in columns (1) and (2), only Age and Education have a statistical significant effect on the probability of being eHealth literate. More specifically, when it comes to the Age effect, there is a negative relationship between eHealth literacy and aging. We find that as the participants grow older, the likelihood of being eHealth literate at the maximum level decreases by 38%, as column (1) indicates. By including other literacy factors (C), namely Computer Literacy and Information Literacy (column 2) the Age effect decreases to 25%. The opposite finding emerges with respect to the Education effect, which is positively related to the eHealth literacy. Particularly, the higher the level of education of the participant, higher is the likelihood of the eHealth maximum level of literacy of the participant, ranging from 70% increase (excluding literacy factors, column 1) to 53% (when literacy factors are included, column 4). The literacy factors are found to greatly affect the eHealth literacy performance of the participants. For example, when we control both literacy factors in column (4), results show that the higher the Computer Literacy and the Information Literacy, the probability of a participant’s maximum level of eHealth literacy increases by 116 and 210%, respectively. The inclusion of these factors slightly decreases the role of the demographic variables, with the former still to pertain their significance.

Next, columns (3) and (4) include only the health life-style (L) factors along with the literacy factors (C). Results demonstrate that all health habit factors carry the expected sign related to their impact on eHealth literacy; however, only physical Exercise is found to be statistically important. If a user does workout more than once a week, his or her eHealth literacy increases by 108% (column 3). In addition, if the participant has high computer and information literacies, then the effect of physical exercise reduces to 64%, as column (4) indicates.

Finally, columns (5) and (6) show estimates of various combinations of all sets of variables. Particularly, last column presents the full-fledged specification with all demographic, life-style, and literacy variables included. As aforementioned, the same variables appear to be statistically significant, maintaining the expected sign according to the theory. For instance, among the demographic factors, the probability of a participant’s eHealth literacy decreases by 23% when the participant ages, while the probability increases by 53% when the participant acquires higher level of education. There is also a positive Marital effect, significant at 10%, on participant’s eHealth literacy; however it’s difficult at this stage of analysis to draw concrete conclusions about the marital effect on eHealth literacy. The reason is that during the movement from one class to the next, one would not be necessarily the case in reality (eg, a divorced person who belongs to class 3 does not necessarily become separated, meaning being member of class 4). Therefore, we cannot compare whether there is an improvement (or deterioration), of any sort, by changing classes, as it is the case with the rest of the variables, which follow an order. Therefore, the marital effect on eHealth literacy requires a marginal effect analysis, which is displayed in Table 6 in this section). With respect to the life-style variables, again physical exercise appears to have a positive and statistical significant effect on a participant’s eHealth literacy, which is about 54%. Literacy factors, relating to computers and information, also document their strong association with eHealth literacy and range from 157% (Computer Literacy) to 207% (Information Literacy).

In total, estimates do not alter either in signor in statistical importance across all specifications of Table 5, and remain robust. Overall, our findings strongly support that the age and education are important contributors to eHealth literacy of an individual. The (negative) effect of age ranges from 23% (column 6) to 37% (column 1), while the (positive) effect of education varies from 70% (column 1) to 53% (column 6). Marital status, only in some cases has a statistically borderline significant role (at 10% level of significance), while the two other remaining demographic variables, that is, income and gender, play no role at all. Physical exercise is the only factor among the life-style set of habit indicators that has a positive and significant effect ranging from 108% (column 3) to 54% (column 6). Smoking and alcohol consumption have no impact on eHealth. In addition, high level of computer and information literacy is positively associated with high probability of eHealth status: 302%–157%, for computer literacy, and 312%–207%, for information literacy. Finally, as diagnostics the later part of Table 4 demonstrates that all specifications have a satisfactory fitness. For the last column, in particular, the fitted values and the actual values are related by 60%.

Next, we performed a marginal effect analysis (Table 6), which captured the effect on maximum eHealth literacy level when an individual changed within variable classes (eg, from low to high income, from primary to high school, etc) at the data means. The analysis was performed for the last column of Table 5, which is the full-fledged specification and is only for the statistical significant variables.

Holding all variables at their mean value, the probability of an individual being eHealth literate at the maximum level is 7% among those aged 15–24 years, 5% among those aged 25–39 years, 4% among those aged 40–54 years, 4% among those aged 55–64 years, 3% among those aged 65–79 years, and 0.3% among those aged above 80 years. For example, as an individual grows old and moves to class 8 (above 80 years old), her probability of being eHealth literate at the maximum level decreases by 2.5% (=[0.028–0.003]*100%). The marginal effect analysis of the effect of various age classes on eHealth literacy confirms the findings from Table 5 that the age effect on eHealth literacy increases as participants become older.

The marginal effect analysis of the marital status on eHealth literacy can be read as follows: the probability of an individual being eHealth literate at the maximum level is about 5% among the singles, 5% among the married, 0.8% among the divorcees, 9% among the separated, and 36% among the widows.

The education effect on eHealth literacy is also consistent with findings from Table 4 as the marginal effects indicate. Overall, higher the level of education of the participant, the larger is the effect on eHealth literacy. For example, when a master holder user (group 7) obtains his PhD and moves to group 8, there is a 7% (=[0.174–0.103]*100%) higher probability in being eHealth literate.

Relating to the impact of physical exercise on eHealth literacy, the marginal effect indicates that someone who is physically active more than once per week (group 1) has a 20% more chance to be eHealth literate.

Finally, when it comes to the technology literacy effects on eHealth literacy, we find that the higher the Computer literacy, higher is the eHealth performance. Particularly, there is not much difference when an individual moves from one computer literacy class to the next higher one. In contrast, there is a two-fold and a four-fold effect when a participant increases his abilities on Information literacy moving from class (1) to (2) and (2) to (3), respectively.

Overall, the marginal effect analysis is in accordance with the odds ratio analysis and further strengthens the robustness of our results.

**Table 3:** Descriptive statistics of all variables.

Variable	Observations	Percentage	Cumulative percentage
eHealth literacy			
Low	189	17.76%	17.76%
Fair	328	30.83%	48.59%
Enough	445	41.82%	90.41%
High	102	9.59%	100.00%
Gender			
Male	477	44.83%	44.83%
Female	587	55.17%	100.00%
Age			
15–24 years	186	17.48%	17.48%
25–39 years	503	47.27%	64.76%
40–54 years	232	21.80%	86.56%
55–64 years	56	5.26%	91.82%
65–79 years	72	6.77%	98.59%
> 80 years	15	1.41%	100.00%
Marital Status			
Single	549	51.60%	51.60%
Married	448	42.11%	93.70%
Divorced	34	3.20%	96.90%
Separated	31	2.91%	99.81%
Widow	2	0.19%	100.00%
Education			
Primary	35	3.29%	3.29%
High school–first 3 years	30	2.82%	6.11%
Technical education	33	3.10%	9.21%
High school—last 3 years	272	25.56%	34.77%
Post-high school—excluding university	51	4.79%	39.57%
University	516	48.50%	88.06%
Masters	106	9.96%	90.03%
PhD	21	1.97%	100.00%
Income			
≤€750	143	13.44%	13.44%
€751–1100	242	22.74%	36.18%
€1101–1450	100	9.40%	45.58%
€1451–1800	164	15.41%	61.00%
€1801–2200	155	14.57%	75.56%
€2201–2800	114	10.71%	86.28%
€2801–3500	94	8.83%	95.11%
>€3500	52	4.89%	100.00%
Smoke			
Nonsmokers	641	60.24%	60.24%
Smokers	423	39.76%	100.00%
Exercise			
Once per week	564	53.01%	53.01%
More than once per week	500	46.99%	100.00%
Alcohol			
Not on a regular basis	829	77.91%	77.91%
On a regular basis	235	22.09%	100.00%
Computer literacy (CL)			
Low	122	11.47%	11.47%
Fair	381	35.81%	47.27%
High	561	52.73%	100.00%
Information literacy (IL)			
Low	160	15.04%	15.04%
Fair	547	51.41%	66.45%
High	357	33.55%	100.00%

**Table 4:** Correlation between eHealth literacy and all independent variables.

Variables	(1)	(2)	(3)	(4)	(5)	(6)	(7)	(8)	(9)	(10)	(11)
1) eHealth	1.00										
(2) Gender	0.01	1.00									
(3) Age	-0.29	-0.02	1.00								
(4) Marital Status	-0.17	0.08	0.57	1.00							
(5) Education	0.41	0.01	-0.22	-0.16	1.00						
(6) Income	0.07	-0.07	0.05	0.11	0.18	1.00					
(7) Smoking	-0.02	-0.07	-0.06	0.01	-0.09	0.04	1.00				
(8) Exercise	0.20	-0.11	-0.22	-0.22	0.11	-0.09	-0.06	1.00			
(9) Alcohol	-0.03	-0.18	-0.01	-0.05	-0.01	-0.02	0.19	0.01	1.00		
(10) Computer literacy	0.46	-0.05	-0.45	-0.31	0.35	0.13	-0.01	0.17	0.04	1.00	
(11) Information literacy	0.45	0.04	-0.17	-0.08	0.27	0.13	-0.06	0.12	-0.09	0.31	1.00

**Table 5:** Logit estimates (odds ratios) of different specifications (maximum level of eHealth literacy is the dependent variable).

	Demographic (D)		Life-style (L)			Full set (X)				
	(1)	(2)	(3)		(4)	(5)		(6)
Gender	1.022 (.854)	1.021 (.863)				1.059 (.635)		1.069 (.591)
Age	0.617 (<.001)	0.752 (<.001)				0.635 (<.001)		0.771 (<.001)
Marital Status	1.081 (.455)	1.169 (.145)				1.121 (.279)		1.201 (.092)
Education	1.698 (<.001)	1.526 (<.001)				1.686 (<.001)		1.530 (<.001)
Income	1.020 (.476)	0.950 (.029)				1.033 (.273)		0.958 (.164)
Smoking			0.956 (.701)		1.070 (.585)	1.024 (.846)		1.157 (.253)
Exercise			2.083 (<.001)		1.638 (<.001)	1.704 (<.001)		1.540 (<.001)
Alcohol			0.877 (.339)		0.926 (.602)	0.868 (.332)		0.929 (.626)
CL		2.584 (<.001)			3.246 (<.001)			2.568 (<.001)
IL		3.102 (<.001)			3.273 (<.001)			3.072 (<.001)
Observations	1064	1064	1064		1064	1064		1064
LR	260.45	506.93	42.88		424.24	280.79		519.79
Pseudo-R2	0.097	0.189	0.016		0.158	0.105		0.194

Note: Numbers in parenthesis are *P* values.

**Table 6:** Marginal Effects Analysis (maximum level of eHealth literacy is the dependent variable).

Variables	Marginal effect	Standard error
Age		
1 (15–24)	0.069	0.012
2 (25–39)	0.052	0.007
3 (40–54)	0.044	0.008
4 (55–64)	0.038	0.011
5 (65–79)	0.028	0.008
6 (>80)	0.003	0.004
Marital Status		
1 (single)	0.046	0.006
2 (married)	0.053	0.007
3 (divorced)	0.008	0.005
4 (separated)	0.095	0.032
5 (widow)	0.364	0.326
Education		
1 (primary)	0.016	0.007
2 (high school—first 3 years)	0.009	0.004
3 (technical education)	0.021	0.008
4 (high school—last 3 years)	0.029	0.005
5 (post-high school—excluding university)	0.025	0.007
6 (university)	0.066	0.008
7 (Masters)	0.103	0.019
8 (PhD)	0.174	0.062
Exercise		
0 (once per week)	0.040	0.006
1 (more than once per week)	0.061	0.008
Computer Literacy		
0 (low)	0.005	0.001
1 (fair)	0.048	0.008
2 (high)	0.078	0.009
Information Literacy		
1 (low)	0.016	0.003
2 (fair)	0.035	0.005
3 (high)	0.120	0.014

## Discussion

Understanding what shapes eHealth in a specific country is particularly important for health policy decision makers and the health care market, as it provides critical information to develop targeted and tailored interventions for relevant patient–consumer segments, and further suggests appropriate strategies for training the health illiterate part of the population. Furthermore, the implementation of eHealth and health information technologies is seen by many as an effective way to address recent concerns about the quality and safety of a health care system, with the rising costs of health care being another major concern that eHealth may help address [26].

For example, the study of Adreassen et al. [21] documents that the use of Internet for health purposes is positively related with youth, higher education, white-collar or no paid job, visits to the general practitioner during the past year, long-term illness or disabilities, and a subjective assessment of one’s own good health. Our findings support the association documented between eHealth literacy, age, and educational level and are in line with many studies that document a similar association [18,20,27]. The study by Rudd et al. [22], along with more recent studies [28,29], further documents the importance of education and age for a person’s eHealth performance. Therefore, the suggestions of another study [30], that is, professional schools should incorporate health literacy into their curricula and areas of competence, seems very reasonable.

The Greek educational system can justify this relationship as Greek students are heavily exposed in new technologies throughout their education, and further enhance the positive age effect demonstrated. In addition, the findings of Watkins and Xie [19] emphasize the need for researchers to develop and assess theory-based interventions applying high-quality research design in eHealth literacy interventions targeting the older population. Baker et al. [17] concluded that higher education is associated with higher use of the Internet for health purposes. A more recent study of Amante et al. [31] has examined various reasons and odds of using the Internet to obtain health information. Cross-country evidence also emphasizes the significance of general literacy level on using information technologies [32,33]. For instance, as literacy skill levels rise, the perceived usefulness of computers, diversity, and intensity of Internet use, and use of computers for task-oriented purposes also rise, even when factors such as age, income, and education levels are taken into account [23].

In contrast, the study of Norman and Skinner [11] has revealed that baseline levels of eHealth literacy are higher among males; age did not predict eHealth literacy scores at any point in time, while overall no significant relationship was found between eHealth literacy and the use of information technology. We do not particularly align studies that find strong association between income and gender with eHealth literacy [29,34,35], as our results do not reveal a strong relation between sex and eHealth literacy, such as the findings of similar studies. In particular, the negative relationship we find can be justified as elderly, who live in the urban regions, may not have access to the Internet.

The link between life-style factors and eHealth literacy is mentioned in the study of Bodie and Dutta [24], but the positive association of these two is not supported. Also, the Neufingerl et al. [36] findings support the low eHealth literacy of smokers; a statement that has not be documented in our research. In contrast, our findings are in line with the Hsu et al. [37] findings, where higher levels of critical eHealth literacy have promoted students’ health status and their practice of multiple positive health behaviors, including eating, exercise, and sleep behaviors. Likewise, the Kontos et al. [33] study finds link between physical activity and eHealth literacy.

Further, our results are in accordance with the studies supporting a positive and strong association between technology literacy and eHealth literacy [24,31,38]. As van Deursen and van Dijk [39] have documented, operational and formal Internet skills are not sufficient when using the Internet for health purposes. Particularly in Greece, limited Internet skills are identified as significant contributing factors to low eHealth literacy [40]. Patients with inadequate health literacy often have poorer health outcomes and increased utilization and costs [41]. The findings of a recent study [42] provide strong evidence that consumer eHealth interventions are of a growing importance in the individual management of health and health behaviors. The latter, is confirmed by the findings of Xie [43] according to which, regardless of the specific learning method used, the eHealth literacy intervention has significantly improved knowledge, skills, and eHealth literacy efficacy from pre- to post-intervention, has been positively perceived by the participants, and led to positive changes in their own health care.

So far, there is thin evidence of theory-based interventions and the eHealth interventions evaluating health outcomes, as the outcome of interest [19]. The incorporation of health literacy assessment into health care information systems and the evaluation of system interventions are recommended by the Institute of Medicine [44] in order to facilitate large-scale studies of the health literacy effects and to improve care by addressing health literacy, respectively. However, a range of access, resources, and skills barriers prevent health care consumers from fully engaging in and benefiting from the spectrum of eHealth interventions such as participating in health discussion forums [42]. Nevertheless, it is feasible to incorporate health literacy screening into clinical assessment, with the next steps being the evaluation of the relation between eHealth literacy and processes and outcomes of care across inpatient and outpatient populations [41].

A handful of studies have demonstrated so far that there is a positive potential with respect to eHealth literacy interventions, though there might be several confounding factors that have contributed to this finding. Although it has been demonstrated that educational level and age play an important role in shaping eHealth literacy level, further research is required in order to evaluate the use of the corresponding questionnaire and the possible ceiling effect. Findings might change in a significant manner if the research addresses only patients (both inpatients and outpatients). Individual motivation, attitudes, and emotional factors are not taken into account, along with the severe Greek economic crisis and its documented association with many health outcomes.

Overall, new measures of eHealth literacy must be developed and evaluated, and eHealth literacy interventions must be incorporated into daily life; therefore, nonfederal funds for eHealth literacy research are further needed particularly in countries under financial crisis, like Greece.

## Conclusion

The advent and development of Internet and its use via various devices, was certainly a disruptive factor in the health provider–consumer (patient) relationship. Further, the Internet has a great potential for disseminating health information to the general public and at the same time is a tool that can be utilized to reach low-income, less educated, minority, and older populations.

Our research aims to study whether certain factors such as demographic, life-style, and types of technology literacy, shape the ability of the individuals in searching, analyzing, and processing information from the Internet in order to address or solve health-related issues.

Using unique survey data of 1064 citizens in Greece for the year 2013, we constructed an eHealth literacy index, based on eight questions, as it has been proposed in the literature, relating a participant’s ability to use the Internet for health issues. Further, we estimated the effect of various factors on an individual’s eHealth activity.

Our results reveal the important role of the age and education effect as well as that of physical exercise on eHealth literacy. Other types of technology literacy, such as computer skills and information obtained from the Internet, further enhance the eHealth performance of an individual having the greatest impact among all others factors.

Our study confirmed that factors influencing eHealth literacy are complex and interdependent. Therefore, more research should be conducted to further explore how these factors may influence one another, taking into consideration specific characteristics of users in various countries.
